# The Measurement of Vital Signs in Pediatric Patients by Lifelight Software in Comparison to the Standard of Care: Protocol for the VISION-Junior Observational Study

**DOI:** 10.2196/58334

**Published:** 2025-03-14

**Authors:** Gauri Misra, Simon Wegerif, Louise Fairlie, Melissa Kapoor, James Fok, Gemma Salt, Jay Halbert, Ian Maconochie, Niall Mullen

**Affiliations:** 1 Mind over Matter Medtech Ltd London United Kingdom; 2 Xim Limited Southampton United Kingdom; 3 South Tyneside and Sunderland NHS Foundation Trust Sunderland United Kingdom; 4 Maidstone and Tunbridge Wells NHS Trust Pembury United Kingdom; 5 Imperial College Healthcare NHS Trust London United Kingdom

**Keywords:** vital signs, remote photoplethysmography, pediatric health assessment, pediatric health monitoring, pediatric, infant, infants, infancy, child, children, Lifelight, software, app, observational study, study protocol, clinical deterioration, COVID-19, SARS-CoV-2, pandemic, telemedicine, medical device, photoplethysmography, eHealth, mobile health, mHealth

## Abstract

**Background:**

Measuring vital signs (VS) is important in potentially unwell children, as a change in VS may indicate a more serious infection than is clinically apparent or herald clinical deterioration. However, currently available methods are not suitable for regular measurement of VS in the home or community setting, and adherence can be poor. The COVID-19 pandemic highlighted a need for the contactless measurement of VS by nonclinical personnel, reinforced by the growing use of telemedicine. The Lifelight app is being developed as a medical device for the contactless measurement of VS using remote photoplethysmography via the camera on smart devices. The VISION-D (Measurement of Vital Signs by Lifelight Software in Comparison to the Standard of Care—Development) and -V (Validation) studies demonstrated the accuracy of the app compared with standard of care (SOC) measurement of blood pressure, pulse rate (PR), and respiratory rate (RR) in adults, supporting certification of Lifelight as a class I Conformité Européenne medical device.

**Objective:**

To support the development of the Lifelight app for pediatric patients, the VISION-Junior study is collecting high-quality data that will be used to develop algorithms for the measurement of VS (PR, RR, and oxygen saturation) in pediatric patients. The accuracy of the app will be assessed against SOC measurements made simultaneously with app measurements.

**Methods:**

The study is recruiting pediatric patients (younger than 18 years of age) attending the Sunderland Royal Hospital pediatric emergency department of the South Tyneside and Sunderland National Health Service Foundation Trust. High-resolution videos of the face (and torso in children younger than 5 years of age) and audio recordings (to explore the value of crying, wheezing, coughing, and other sounds in predicting illness) are made using the Lifelight Data Collect app. VS are measured simultaneously using SOC methods (finger clip sensor for PR and oxygen saturation; manual counting of RR). Feedback from patients, parents, carers, and nurses who use Lifelight is collected via questionnaires. Anticipated recruitment is 500 participants, with subtargets for age, sex, and skin tone distribution (Fitzpatrick 6-point scale). Early data will be used to refine the algorithms. A separate dataset will be retained to test the performance of the app against predefined targets.

**Results:**

The study started on June 12, 2023, and reached its recruitment target (n=532) in April 2024 after extending the deadline. Algorithm refinement is in progress, after which the performance of Lifelight will be compared with the SOC measurement of VS. The analyses are expected to be completed by mid-August 2024.

**Conclusions:**

Data collected in this study will be used to develop and assess the accuracy of the app for the measurement of VS in pediatric patients of all ages.

**Trial Registration:**

ClinicalTrials.gov NCT05850013; https://clinicaltrials.gov/study/NCT05850013

**International Registered Report Identifier (IRRID):**

DERR1-10.2196/58334

## Introduction

Measurement of vital signs (VS) is an essential part of clinical evaluation, providing important information about a patient’s health; importantly, a change in VS may herald a deterioration in clinical condition [1] and may precede the emergence of physical symptoms during the early stages of an infection [2]. High pulse rate (PR) and respiratory rate (RR) have been shown to predict hospital admission in children [3]. Infections such as influenza, scarlet fever, and chickenpox are common causes of morbidity and mortality in immunocompromised children [4,5]. Three-quarters of children who develop severe sepsis from infection have a comorbid condition such as asthma, diabetes, cystic fibrosis, or cancer. Sepsis carries a mortality rate of almost 50% in the most vulnerable children [6]. Treatment of infection in children can be expensive, especially if intensive care is required.

Given that monitoring of VS is important in clinically vulnerable children to aid early detection of infection [7], a robust method that parents can use at home to measure VS is likely to be beneficial and would support remote initial assessment [8]. The measurement of VS is time-consuming and requires trained staff and multiple pieces of equipment, some of which need regular calibration. Accurate measurement of blood pressure (BP) using a sphygmomanometer requires the use of the correct size cuff for the patient. The physical contact of equipment can be uncomfortable and distressing for patients, particularly those with learning disabilities. Stress can affect BP, PR, and RR, such that the measurements may not accurately reflect the patient’s state of health [7]. Standard of care (SOC) medical equipment is not suitable for regular measurement of VS in the home or community setting. Adherence to frequent VS monitoring using traditional technologies is low because of the inconvenience and stigmatizing nature of specialist hardware equipment (eg, thermometers, BP cuffs, and finger-clip oximeters) [7]. The COVID-19 pandemic has also highlighted the need for methods suitable for the remote or contactless measurement of VS that can be operated by people without specific medical training. The growing use of telemedicine since the pandemic also points to the need for easy but accurate measurement of VS.

Photoplethysmography (PPG) is an optical measurement technique that records changes in the light reflected from the skin surface due to volumetric changes in the facial blood vessels; small variations in perfusion provide valuable information about the cardiovascular system [9]. PPG has been used to measure PR [10,11], oxygen saturation [12], BP [13,14], and RR [10,15] and to detect atrial fibrillation [16].

Lifelight is an app being developed for the contactless measurement of VS using remote PPG via the camera on smart devices such as phones and tablets. The app captures the average color of regions of interest on the face 30 times every second for 60 seconds and sends these as red, green, and blue color values to a central server for processing, yielding VS values. The VISION-D (Measurement of Vital Signs by Lifelight Software in Comparison to the Standard of Care—Development) study in 2018-2019 measured VS in 8585 patients and healthy volunteers simultaneously using the app and SOC methods. The data were used for machine learning to develop the algorithms used to calculate VS. The subsequent VISION-V (Validation) study demonstrated the accuracy of the app compared with SOC methods for measuring PR, RR, and diastolic BP [17], providing the basis for the current Class I Conformité Européenne (CE) mark [18]. The app also received ISO 13485:2016 certification in August 2023 [19], and the accuracy of the app for measuring PR and BP against ISO standards in adults with stage 1 hypertension has recently been reported [20]. The VISION-MD (multisite development) study evaluates the app versus SOC in patients with a wide range of clinically relevant VS values, including critically ill patients, and across the full range of skin tones [21]. In VISION-MD, high-resolution full-face videos of participants’ faces have been recorded, which has proved instrumental in improving the accuracy of VS measurement compared with the VISION-D and -V studies.

The VISION-Junior study explores the measurement of VS using a Lifelight app developed for pediatric patients (aged 18 years or younger) attending the pediatric emergency department. As in VISION-MD in adults, high-resolution full-face videos will be recorded. In younger children (younger than 5 years of age), the video will also include the torso to increase the surface area from which the signal is recorded, and because the torso is likely to move less than the face. Audio data will also be recorded, as crying, wheezing, coughing, and other sounds can also predict illness in children [22,23].

The aim of the VISION-Junior study is to assess whether the app can be used to accurately measure VS in pediatric patients. This protocol describes the collection of data that will initially be used to develop the algorithms for the measurement of VS in children. Later data will be used to assess the accuracy of the app across the range of VS values encountered in children of different ages. The objectives are as follows.

The primary objective is to collect data to support the development of the app for the accurate measurement of PR, RR, and oxygen saturation in pediatric patients and to assess the accuracy of measurements against SOC methods. Secondary objectives are: (1) to assess—using the collected PPG data—whether other (direct or indirect) measurements can be made using the app, including temperature, BP, PR variability, and audio analysis; (2) to evaluate the impact of subject-specific variables on the PPG VS measurements (eg, medication, cosmetics, facial or body hair, and skin tone); (3) to obtain data to understand patient and parent or guardian or carer habits and preferences for VS measurement using questionnaires (Multimedia Appendices 1 and 2); and (4) to assess the usability of Lifelight Junior for measuring VS in pediatric patients through questionnaires completed by participating health care professionals (Multimedia Appendix 3).

Most of the data collected in the study will be used to train the app algorithms to measure VS in pediatric patients. The remaining data will be kept separate and used to assess the accuracy of the algorithms at regular intervals during the data collection period. The study delivery process will be responsive to this learning curve to increase the likelihood that the predetermined accuracy target for each VS estimation is achieved by the study end.

## Methods

The design and protocol for this study have been informed by the VISION-MD study [21] and earlier VISION studies [17].

### Participants and Recruitment

Approximately 500 pediatric patients are being recruited at the Sunderland Royal Hospital pediatric emergency department of the South Tyneside and Sunderland National Health Service (NHS) Foundation Trust. All pediatric patients (younger than 18 years of age) attending the pediatric emergency department are potentially eligible for the study. The exclusion criteria are critical illness (as judged by the treating physician), including unconscious patients, patients who were noncompliant in terms of excessive movement during the VS measurements, and patients whose parents, guardians, or carers do not speak English (required to give informed consent). The study aims to recruit according to age, sex, Fitzpatrick skin tone, and medical history criteria, as subsequently described.

The research nurse approaches the parents, guardians, or carers of potential participants identified by the clinical team about taking part in the study. Potential participants aged 17 or 18 years or the parents, guardians, or carers of younger children are provided with an ethics-approved information sheet explaining the study aims, what is involved, and the requirements for participation. Younger children also watch ethics-approved age-appropriate videos (for ages 5-10 and 11-16 years) explaining the study on a tablet device (Multimedia Appendices 4 and 5).

The information sheet and videos were developed in collaboration with the Young People Advisory Group North England and the South Tyneside and Sunderland NHS Foundation Trust Young Persons Group. These groups also provided feedback on the design of the study, consent/assent forms, and other study documents at 2 in-person meetings.

### Study Procedures

Demographic data (age, sex, ethnicity, skin tone [Fitzpatrick 6-point scale [24] ranging from type 1 for skin that always burns to type 6 for skin that never burns]) are recorded on an electronic case report form using the Castor electronic platform. Medical history questions are limited to the presence or absence of conditions that might affect skin perfusion and pigmentation, cardiovascular processes, and any prescription medicines for these conditions.

The study staff records a set of premeasurement observations and the presence or absence of sweat on the participant’s face; any facial hair on the cheeks; tattoos, jewelry, birthmarks, scars, or other features on the face; the use of foundation or concealer; and the position of the participant (seated, prone, supine or lying on one side).

Patients may also be recruited into 1 of 3 subprotocols depending on premeasurement observations (Table 1).

The study staff ensures that participants have been at rest for at least 10 minutes before VS measurement starts (which can include the period of data collection described previously).

**Table 1:** Subprotocols.

	Subprotocol	Description
1	Blood pressure measurement	Blood pressure will be measured at the same time as other vital signs using standard of care equipment (sphygmomanometer) in participants who are able to cooperate and stay relatively still
2	Electrocardiogram	A 3-lead electrocardiogram will be recorded to assess heart rate variability in a representative subset of participants (based on age, skin tone, and illness; see Table 3)
3	Questionnaire	Guardians, parents, or carers and children will be asked to complete a questionnaire about their preferences for Lifelight-type technologies versus standard of care methods for measuring vital signs

In each study session, VS is measured as per the subprotocol using the SOC equipment, while simultaneously capturing a video of the participant’s face using the Lifelight Data Collect app running on a tablet (standard iPad). This app is a research tool that captures and uploads video data to secure cloud storage for research purposes only and is distinct from the CE-marked Lifelight medical device developed from data collected from previous studies. The Data Collect app does not display any VS estimates and is not used for clinical interpretation or analysis.

In each study session, at least 2 VS (PR, oxygen saturation, RR) and temperature are measured twice over a 60-second period using SOC equipment and methods.

A standard clinical finger clip sensor for the measurement of oxygen saturation and PR is placed on a finger. Oxygen saturation and PR are measured at 0, 30, and 60 seconds of the recording period and averaged.

RR is determined by counting chest rises throughout the 60-second period. The nurse may place their hand on the participant’s chest to improve the accuracy of counting while being mindful not to obscure the camera’s line of sight.

At the same time, a video of the participant’s face (and torso in children younger than 5 years of age) and audio is captured for up to 3 minutes using the Data Collect app running on a tablet positioned approximately 1 meter away and angled toward the participant’s face. Background luminosity is measured automatically. Recording is started and stopped by the research nurse via the iPad.

A maximum of 3 attempts at recording measurements is made. Once measurements have been completed, the study staff fill in postmeasurement questions relating to how much the participant moved, their position, whether they were wearing glasses, any hairstyle or other item (eg, face covering) that obscured any part of their face during the recording, and whether the app reported “face not found” at any point during the recording.

A questionnaire is also available to garner feedback on the technology from the clinical user’s point of view (ie, the nurses who take the VS measurements). Questionnaire data are fully anonymized and recorded without any identifiable information.

Each study session, including approaching the participant, obtaining consent, and performing the study procedure takes about 30 minutes. Participants were not followed up after the VS measurements but continued to receive clinical care as appropriate for their condition.

### Subprotocols

**Figure 1:**
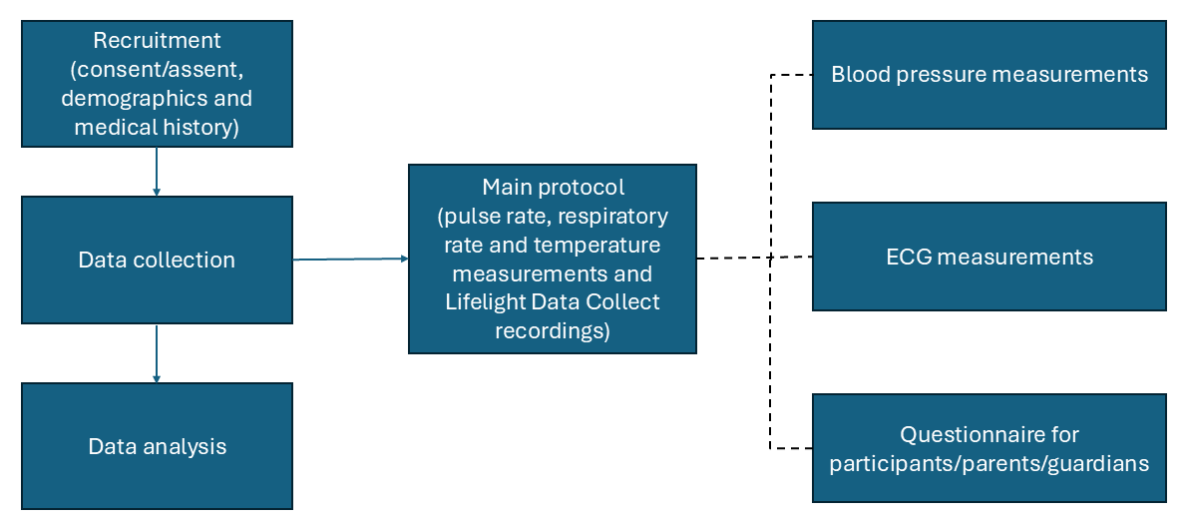
Study flow and data collection. BP: blood pressure; ECG: electrocardiogram.

Participants are recruited into 3 subprotocols as appropriate (Table 1). Subprotocols 1 and 2 may be run concurrently with the main protocol if the research nurse considers it practical to also record BP and an electrocardiogram (Figure 1).

### Ethical Considerations

The study is being performed in accordance with the spirit and the letter of the Declaration of Helsinki, the Good Clinical Practice Guidelines, and the protocol and applicable local regulatory requirements and laws. The VISION-Junior protocol was approved by the Health Research Authority and Health and Care Research Wales (IRAS reference 321956) and the Newcastle North Tyneside Research Ethics Committee on March 30, 2023. Informed consent is obtained electronically using Castor eConsent, a secure NHS-compliant platform. Informed consent is provided by participants aged 16 years and older and by the parent, guardian, or carer of younger children. Children younger than 16 years of age are also given the opportunity to sign an assent form. As the study is noninterventional, participants will not receive financial compensation for participation but may be offered small incentives such as stickers or certificates. Participation in the study is entirely voluntary, and participants may withdraw from the study at any time. Specific, explicit written consent was obtained from all individuals featured in the videos provided in Multimedia appendices 4 and 5, permitting use for publication purposes.

### Privacy and Data Collection

Each study participant is assigned a unique sequential patient identifier; no identifiable data are stored. All documents are stored securely and can only be accessed by study staff and authorized personnel. The code linking the patient identifier to their personal information is kept within the hospital study site and can only be accessed by the research team within that site.

For each reading, a high-quality video of the face (and torso in young children) and an audio file are saved to the internal storage of the iPad in encrypted form and transmitted directly to the sponsor’s NHS-compliant cloud server. Files were automatically deleted following upload to the server.

All data collected about participants during the study will be kept strictly confidential and handled according to the General Data Protection Regulations. Videos and audio recordings collected during the study constitute personal data as it may be possible to identify participants. Full-resolution, full-face, or torso video data are required to develop Lifelight Junior into a clinically useful device; the analysis cannot be performed using blurred videos. The data collected for research purposes only (not clinical care) are processed within the legitimate interests of Xim Ltd.

### Data Handling

Analysis of the videos uses the encrypted files, which, in most cases, are processed automatically within a secure cloud infrastructure (ie, without any person viewing the videos); in rare cases, it may be necessary to analyze a video manually if the recording is not as expected. The analysis results in anonymized aggregate datasets. No decrypted files are stored at any point in the processing. The electronic case report forms are stored and managed using the Castor electronic cloud.

### Performance Targets

While Lifelight is certified as a class I CE medical device [18], the performance of Lifelight Junior needs to be appropriate for routine clinical practice in pediatric patients. Table 2 lists the performance targets for Lifelight Junior for measuring VS in pediatric patients; training data collected during VISION-Junior will support progress toward these targets.

**Table 2:** Performance targets in VISION-Junior.

Vital sign	Performance target	Reference
Pulse rate	Root mean square error ≤5 beats per minute	[25]
Respiratory rate	Maximum error of 5 breaths per minute for 100% of measurements	Minimum standard met by all approved respiratory monitors
Oxygen saturation	Maximum error tolerance 4%	[26]

### Sample Size

An estimated 500 pediatric patients (18 years old or younger) will be recruited into the study. This sample size is informed by the previous VISION-D, VISION-MD, and VISION-Acute studies [17,21]. However, the sample size cannot be formally calculated because it depends on the incremental improvement in the performance of the app achieved through machine learning using the training data generated early in the study. The split between training and testing data will be determined during the study according to the quality of the data collected. The study management team monitors the progress of data collection and accuracy and updates the study teams weekly. The study will continue until the accuracy of the app for measuring VS in pediatric patients is sufficient for various clinical use cases.

In addition, recruitment targets according to age, sex, Fitzpatrick skin tone, and medical history have been defined, as shown in Table 3, to ensure accuracy in a broad range of clinically relevant patients.

**Table 3:** Demographic and medical history recruitment targets.

	Targets
Age	≥12% aged 0-1, 1-2, 2-5, 5-11, 11-14, 14-18 years
Sex	≥40% male; ≥40% female
Skin tone	15% of participants will have Fitzpatrick skin tones 4, 5, or 6 (this is not a hard target as achieving this figure depends on the demographic diversity of the study region)
Medical history	≥40% in each of the following categories: Febrile (≥38 °C) Afebrile Ill (as judged by the clinical staff); to ensure a spread of conditions, no more than 50% of recruited patients will have an acute respiratory illness (eg, bronchiolitis, asthma, virus-induced wheeze, pneumonia, and croup) Well, including pediatric patients with a minor injury

### Data Analysis

All statistical analyses will be performed using Python (Python Software Foundation). Training data will be used for machine learning to improve the performance of Lifelight Junior for measuring VS in pediatric patients. Appropriate machine learning models will be developed according to the data collected. This is likely to include signal processing and measurement algorithms for PR and RR, and multilayer neural network-based approaches for BP, similar to those used in the adult version of the app.

The separate set of test data will subsequently be used to determine the performance of the app for measuring VS in pediatric patients. Correspondence between VS measurements predicted by the app and the measurements taken manually using SOC methods will be compared using regression coefficients, mean error, and SD, and compared against the performance targets set out in Table 2. An accuracy target will be considered met if the mean error and SD for the app measurements are at least equal to the target (Table 2). Heat maps or scatter plots will also be developed.

Linear regression will be used to assess the impact of skin tone on the accuracy of the app for measuring each VS, using the Fitzpatrick skin tones as the explanatory variable.

Quantitative data from the questionnaires will be summarized. Thematic analysis will be used to analyze free-text responses to identify perceptions of the app and variations in the way patients, their parents or carers, and health care professionals interact with it and any challenges they encounter.

All analyses will be completed per protocol since there is no intention to treat. There will be no imputation of missing or implausible data, and any missing, implausible, or problematic readings will be excluded from analysis.

## Results

The study started on June 12, 2023, and as of December 20, 2023, had recruited 303 participants. The study was extended until the end of March 2024 to enable the target recruitment (500) to be met; 532 participants had been recruited as of April 5, 2024.

Data are currently being used for machine learning to refine the algorithms to improve clinical accuracy. We anticipate that final analyses to determine the performance of the app against the targets set out in Table 2 will be complete by mid-August 2024. The performance of the app will be determined across the range of VS values encountered in children in clinical practice by comparing the VS values recorded using PPG versus standard methods.

## Discussion

### Principal Findings

This paper reports the protocol for obtaining data to develop algorithms for the measurement of VS in children using the app, and to evaluate the performance of the app against SOC measurement of VS in pediatric patients of all ages.

Monitoring of VS in children is challenging because existing methods (eg, BP measurement via an arterial line or cuff; PR and RR via electrodes, adhesive pulse oximeters, and adhesive temperature probes and thermometers) are either invasive or contact-based and can cause discomfort, pain or distress, may disturb sleep, and may damage paper-thin skin, potentially increasing the risk of infection. Frequent VS measurement using different techniques is also resource-intensive. The app being developed will provide contactless noninvasive measurement of VS.

Data collected in this and other studies will be used to train algorithms to develop the app for the measurement of VS in pediatric patients. The app aims to provide a risk-measured way for nonexperts to measure VS quickly and nonintrusively, and therefore, detect any early signs of deteriorating health or infection. This will make it easier for clinically vulnerable or immunocompromised children to attend school and interact with their peer groups, meeting their mental, physical, social, and educational needs, whilst providing reassurance to parents, guardians, or carers. Early detection of infection before any significant clinical deterioration occurs means that treatment can start sooner, improving the likelihood of a full recovery [27] whilst also potentially avoiding hospital admission and intensive care, which are expensive for the child’s family (lost income, expenses) and health care providers. Lifelight can also be an effective tool for at-home monitoring and triage, reducing the burden on carers, emergency services, and hospitals. It may also be useful in nonspecialist health care settings such as primary care and prehospital care where specialist pediatric equipment is not available. Several other devices for the measurement of VS are currently being developed but all require physical contact, whether through a finger, the wrist, or the chest, and only one (Biospectral) is able to measure BP.

### Limitations

This research may have some limitations. BP is inherently more complex to measure than PR and RR, in terms of the data form and machine learning and because reference measurements are less accurate. As with any recording device, signal quality may be compromised if the participant moves excessively or if light levels are insufficient. However, we expect the use of high-resolution video recording to overcome such issues. In addition, measurements can be easily repeated if a recording is of poor quality.

Skin type is a potential source of error with PPG devices, as melanin absorbs green light, potentially increasing errors in measurements in dark-compared with light-skinned individuals [24]. However, we have already shown that skin type does not affect the accuracy of Lifelight [17]. Although the Fitzpatrick Skin Type Scale is the current gold standard [24], its use has been criticized because of racial bias, weak correlation with skin color, and broad within-group variations in skin tone. Spectrocolorimetry, which uses multiple variables to categorize skin tone objectively, has been proposed as an alternative [24], which may be incorporated into later studies to confirm our findings.

### Conclusions

The results of this study will be disseminated via submissions to relevant journals and conferences. The study team will explore different avenues and formats for the dissemination of the study findings with the Young People Advisory Groups and other groups to ensure a wide public audience, for example, through coproduced public talks, studies in community communications, and social media.

## Appendix

Multimedia Appendix 1 Questionnaire to collect data from participants in the VISION-Junior observational pediatric study in order to improve the usability of the Lifelight app.

Multimedia Appendix 2 Questionnaire to collect data from parents/guardians/carers of participants in the VISION-Junior observational pediatric study in order to improve the usability of the Lifelight app.

Multimedia Appendix 3 Questionnaire to collect data from health care practitioners conducting the VISION-Junior observational pediatric study in order to improve the usability of the Lifelight app.

Multimedia Appendix 4 Participant information video (ages 5-11) explaining the VISION-Junior observational pediatric study process to prospective participants before consent.

Multimedia Appendix 5 Participant information video (≥11 years old) explaining the VISION-Junior observational pediatric study process to prospective participants before consent.

## Abbreviations

BP: blood pressure
CE: Conformité Européenne
NHS: National Health Service
PPG: photoplethysmography
PR: pulse rate
RR: respiratory rate
SOC: standard of care
VISION-D: Measurement of Vital Signs by Lifelight Software in Comparison to the Standard of Care—Development
VISION-V: Measurement of Vital Signs by Lifelight Software in Comparison to the Standard of Care—Validation
VISION-MD: Measurement of Vital Signs by Lifelight Software in Comparison to the Standard of Care—multisite development
VS: vital signs

## Data Availability

The datasets generated or analyzed during this study are not publicly available due to their commercially sensitive nature, as mandated by Xim Limited’s grant funders and investors, but are available from the corresponding author upon reasonable request.
