# Supplementary material for: The Measurement of Vital Signs in Pediatric Patients by Lifelight Software in Comparison to the Standard of Care: Protocol for the VISION-Junior Observational Study
Source: JMIR Res Protoc. 2025 Mar 14;14:e58334. doi: 10.2196/58334 (PMC11953603; doi:10.2196/58334)
Supplement: Multimedia Appendix 1 [file resprot_v14i1e58334_app1.docx]

# The measurement of Vital Signs in children by Lifelight^®^ software

# in comparison to the standard of care

# The VISION-Junior study Participant questionnaire

Please can you answer a few quick questions about Lifelight? This will really help us make our invention better! Your participation will help us change the future! Do not worry, all your answers will be safe with us, and no one will get to know your identity through them.

- How did you find Lifelight?
- Would you prefer it if doctors and nurses checked your health using Lifelight or using the normal equipment the doctor/nurse generally uses such as a blood pressure cuff, thermometer and finger-clip? (Lifelight/Normal Equipment)
- Why do you say this?

Thank you for your time
