# Supplementary material for: The Measurement of Vital Signs in Pediatric Patients by Lifelight Software in Comparison to the Standard of Care: Protocol for the VISION-Junior Observational Study
Source: JMIR Res Protoc. 2025 Mar 14;14:e58334. doi: 10.2196/58334 (PMC11953603; doi:10.2196/58334)
Supplement: Multimedia Appendix 2 [file resprot_v14i1e58334_app2.docx]

# The measurement of Vital Signs in children by Lifelight^®^ software

# in comparison to the standard of care

# The VISION-Junior study Parent/guardian questionnaire

We would be grateful if you could please answer the following questions to help us improve the usability of the Lifelight® app. Any information you provide is anonymous and kept securely.

- How did you find the Lifelight® app?
- Would you prefer it if your child’s/ward’s vital signs were measured using Lifelight® or using the standard equipment (blood pressure cuff, thermometer, finger-clip pulse oximeter)?
- What are your thoughts on the idea that Lifelight® could be used as the main way to measure heart rate, respiratory rate, temperature, blood pressure and oxygen saturation in future?
- How do you think your ward/child would respond?
- Do you have a blood pressure monitor at home? If yes, how often have you used it? Why did you use it?
- Do you have a finger-clip oximeter at home? If yes, how often do you use it? Why did you use it?
- Do you have a thermometer at home? If yes, how often do you use it? Why did you use it? What type of thermometer is it (armpit, ear, mouth, forehead, etc)?
- Do you know how accurate your devices are?
- If you had Lifelight® at home, do you think you would measure your child/ward’s heart rate, respiratory rate, oxygen saturation, blood pressure or temperature more often?
- Do you think Lifelight® could be useful for home monitoring, especially in cases of critical/debilitating illnesses?

Thank you for your time
