# Supplementary material for: The Measurement of Vital Signs in Pediatric Patients by Lifelight Software in Comparison to the Standard of Care: Protocol for the VISION-Junior Observational Study
Source: JMIR Res Protoc. 2025 Mar 14;14:e58334. doi: 10.2196/58334 (PMC11953603; doi:10.2196/58334)
Supplement: Multimedia Appendix 3 [file resprot_v14i1e58334_app3.docx]

# **The measurement of Vital Signs in children by Lifelight^®^ software**

# **in comparison to the standard of care**

# **The VISION-Junior study** **Healthcare professional questionnaire**

We would be grateful if you could please answer the following questions to help us improve the usability of the Lifelight® app. Any information you provide is anonymous and kept securely.

- How did you find operating the Lifelight® app?
- Do you think patients will prefer having their blood pressure measured using Lifelight® or using the blood pressure cuff? Do you think this preference will be stronger in adult patients or child patients?
- What are your thoughts on the idea that Lifelight® could be used as the main way to measure blood pressure, heart rate, respiratory rate and oxygen saturation in future? How do you think patients would respond?

Thank you for your time
